# Rhythmic neural activity is comodulated with short-term gait modifications during first-time use of a dummy prosthesis: a pilot study

**DOI:** 10.1186/s12984-020-00761-8

**Published:** 2020-10-08

**Authors:** Vera G. M. Kooiman, Helco G. van Keeken, Natasha M. Maurits, Vivian Weerdesteyn, Teodoro Solis-Escalante

**Affiliations:** 1 Orthopaedic Research Laboratory, Radboud University Medical Center, (611) P.O. Box 9101, NL-6500 HB Nijmegen, The Netherlands; 2 Department of Rehabilitation, Donders Institute for Brain, Cognition and Behavior, Radboud University Medical Centre, (898) P.O. Box 9101, NL-6500 HB Nijmegen, The Netherlands; 3 Center for Human Movement Sciences, University of Groningen, University Medical Center Groningen, UMCG Sector F, FA 23, P.O. Box 196, NL-9700 AD Groningen, The Netherlands; 4 Department of Neurology, University of Groningen, University Medical Center Groningen, Hanzeplein 1, P.O. Box 30.001, Groningen, The Netherlands; 5 Sint Maartenskliniek, Research & Rehabilitation, P.O. Box 9011, NL-6500 GM Nijmegen, The Netherlands

**Keywords:** Gait, Prosthesis, EEG, Gait modifications, Mobile brain/body imaging

## Abstract

**Background:**

After transfemoral amputation, many hours of practice are needed to re-learn walking with a prosthesis. The long adaptation process that consolidates a novel gait pattern seems to depend on cerebellar function for reinforcement of specific gait modifications, but the precise, step-by-step gait modifications (e.g., foot placement) most likely rely on top-down commands from the brainstem and cerebral cortex. The aim of this study was to identify, in able-bodied individuals, the specific modulations of cortical rhythms that accompany short-term gait modifications during first-time use of a dummy prosthesis.

**Methods:**

Fourteen naïve participants walked on a treadmill without (one block, 4 min) and with a dummy prosthesis (three blocks, 3 × 4 min), while ground reaction forces and 32-channel EEG were recorded. Gait cycle duration, stance phase duration, step width, maximal ground reaction force and, ground reaction force trace over time were measured to identify gait modifications. Independent component analysis of EEG data isolated brain-related activity from distinct anatomical sources. The source-level data were segmented into gait cycles and analyzed in the time–frequency domain to reveal relative enhancement or suppression of intrinsic cortical oscillations. Differences between walking conditions were evaluated with one-way ANOVA and post-hoc testing (α = 0.05).

**Results:**

Immediate modifications occurred in the gait parameters when participants were introduced to the dummy prosthesis. Except for gait cycle duration, these modifications remained throughout the duration of the experimental session. Power modulations of the theta, mu, beta, and gamma rhythms, of sources presumably from the fronto-central and the parietal cortices, were found across the experimental session. Significant power modulations of the theta, beta, and gamma rhythms within the gait cycle were predominately found around the heel strike of both feet and the swing phase of the right (prosthetic) leg.

**Conclusions:**

The modulations of cortical activity could be related to whole-body coordination, including the swing phase and placing of the prosthesis, and the bodyweight transfer between legs and arms. Reduced power modulation of the gamma rhythm within the experimental session may indicate initial motor memories being formed. Better understanding of the sensorimotor processes behind gait modifications may inform the development of neurofeedback strategies to assist gait rehabilitation.

## Background

In everyday life, walking requires flexible adjustments of a stereotypical gait pattern according to varying environmental conditions and task demands. In able-bodied individuals, years of walking practice allow for seamless gait adjustments with limited conscious effort [1, 2]. However, with a transfemoral amputation, part of the locomotor system is lost, gait capacity is drastically reduced, and simple gait adjustments become burdensome. This is particularly the case for those who use a mechanical knee prosthesis, where correct foot placement and the initiation of the swing phase is crucial to prevent knee buckling or stumbles during walking [3]. An incorrect placement of the foot can create a flexion moment on the knee, causing the knee to buckle during initial stance phase, whereas a correct placement of the prosthetic foot will lock the knee to ensure it can be safely loaded during the stance phase. To initiate the swing phase, the extension moment needs to be changed into a flexion moment ensuring enough clearance between the prosthetic foot and the ground to avoid a stumble or trip. These implications cause gait to be more physically and cognitively demanding for individuals with a transfemoral amputation [4–7] and requires a long rehabilitation process to fully comprehend this skill.

During gait rehabilitation with a lower limb prosthesis, many hours of practice are needed to permanently adapt the gait pattern, taking into account the loss of direct control over knee and ankle joints, the loss of sensory feedback from the lower leg, and the dynamics of the lower limb prosthesis. The long adaptation process that consolidates a novel gait pattern gradually occurs through iterative short-term modifications of the stereotypical gait pattern [8, 9]. The acquisition and consolidation of a novel gait pattern seem to depend on cerebellar function for reinforcement of specific gait modifications [8, 10], but the precise, step-by-step gait modifications (e.g., foot placement) most likely rely on top-down commands from the brainstem and cerebral cortex [11, 12].

Studies on mobile brain/body imaging (MoBI) demonstrate modulations of cortical rhythms linked to dynamic gait modifications. Mu and beta rhythms, source-localized from scalp-level recordings to the premotor and parietal cortices, are suppressed during volitional gait cycle modification during treadmill walking [13]. Since the suppression of mu and beta rhythms typically accompanies both the preparation and execution of voluntary movements [14], the authors suggested that their results could reflect the activity of a premotor-parietal cortical network involved in the preparation and execution of gait modifications [13]. Beta rhythms are enhanced, source-localized from scalp-level recordings to the prefrontal cortex, during step-shortening (compared to step-lengthening) in auditory cue-guided treadmill walking [15]. The enhancement of the beta rhythm was linked to motor inhibition processes [15], consistent with the presumed functional role of the beta rhythm [14, 16]. Together, this implies a prominent role of mu and beta rhythms for dynamic gait modifications. Also, power modulations of the beta rhythm, source-localized from scalp-level recordings to the premotor cortices, could be related to postural stabilization during steady-state treadmill walking [17]. Other studies have shown modulations of prefrontal, premotor, and sensorimotor mu and beta rhythms, as well as metabolic changes in prefrontal and sensorimotor cortices, during visually guided and precision stepping (see [18] for a recent review). In addition, recent studies have shown a causal effect (established by means of directed coherence and Granger causality analyses) of multiple cortical rhythms from many different cortical areas on coordinated muscle activity during treadmill walking [19, 20], and provide compelling evidence for the direct involvement of the cerebral cortex in step-by-step modifications of the stereotypical gait pattern. These studies show that modulations of the cortical mu and beta rhythms, presumably from prefrontal, premotor, and parietal cortices, are related to the dynamic gait modifications. Therefore, the mu and beta rhythms may reflect cortical mechanisms for top-down control of gait.

The goal of this study was to identify, in able-bodied individuals, the specific modulations of cortical rhythms that accompany short-term gait modifications during first-time use of a dummy mechanical knee prosthesis. Our pilot study focused on investigating the first-time use of a prosthesis in able-bodied individuals, because an experiment on the immediate use of a prosthesis following an amputation would lead to additional undesirable burden to the patients. With the use of a dummy prosthesis, it is possible to simulate the changes to the locomotor system that come with a transfemoral amputation, without affecting the central and peripheral nervous system. We hypothesized that mu and beta rhythms from prefrontal, sensorimotor, and parietal cortices would reflect the immediate gait modifications related to the first-time use of the dummy prosthesis. It was expected that the power of the mu and beta rhythms would be reduced during steady-state walking with the dummy prosthesis, relative to walking without the dummy prosthesis, thus indicating a stronger cortical activation and top-down control of gait. Such modulations may reflect initial mechanisms of long-term modifications (i.e., possibly leading to permanent adaptation) of the gait pattern within a single experimental session (approximately 12 min of practice). We expected to find stronger effects (i.e., power modulations) during early use of the dummy prosthesis that weaken with repeated use of the dummy prosthesis. Better understanding of the sensorimotor processes behind gait modifications may inform the development of novel neurofeedback strategies to assist gait rehabilitation.

## Methods

### Participants

Fourteen able-bodied male individuals participated in this study (12 right-footed). All individuals provided written informed consent prior to participation in the experiment. The datasets from two participants had to be excluded due to poor quality of their EEG signals (after visual inspection and epoch rejection). Thus, data from 12 participants (21 ± 2 years, 83 ± 12 kg, 186 ± 6 cm) were analyzed. The experimental procedure was approved by the Ethical Committee for the department of Human Movement Sciences of the University of Groningen. The procedures complied with the guidelines defined in the Declaration of Helsinki [21].

### Experimental procedure

**Fig. 1:**
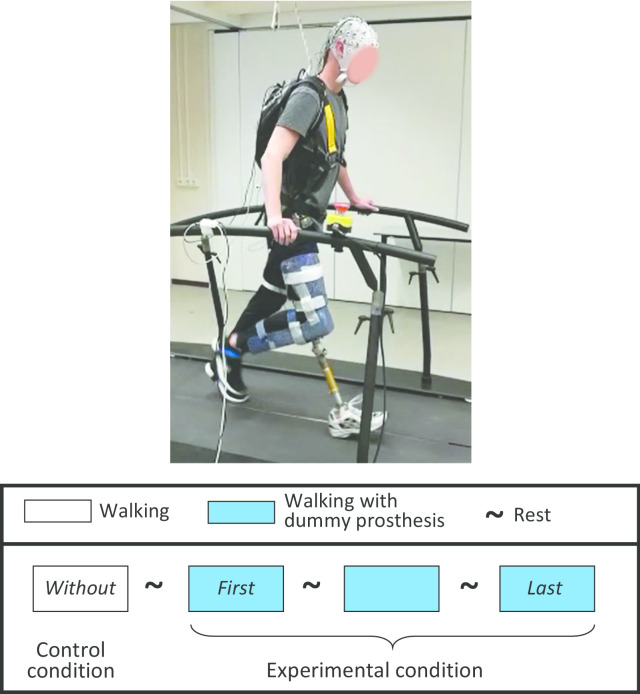
The experimental setup during the measurements and a schematic overview of the timing of the procedure. Resting period was 4 min

Participants walked on a treadmill with and without a dummy mechanical knee prosthesis that simulates walking with a transfemoral prosthesis [3, 22, 23]. None of the participants had previous experience with the dummy prosthesis, and no specific instructions were given with respect to the use of the prosthesis or to the usage of the handrails during walking. The walking speed was kept constant across all participants at 0.9 m/s. The walking speed was selected based on energy efficiency and the average walking speed of people with a transfemoral amputation [24, 25]. All participants completed four blocks of 4-min treadmill walking with resting periods of 4 min in between (see Fig. 1).

In the first block, participants walked on the treadmill without the dummy prosthesis. After the first block, the dummy prosthesis was fitted to the right leg of the participants. Participants were not allowed to practice walking on the prosthesis before the measurement, but were allowed to flex, extend and support themselves with the knee of the dummy prosthesis, to familiarize themselves with the mechanism of the prosthesis. In the second, third, and fourth blocks, the participants used the dummy mechanical knee prosthesis to walk. Figure 1 illustrates the experimental setup and the timing of the procedure.

### Data acquisition

Multi-channel electroencephalogram (EEG), ground reaction forces (GRF), and center of pressure (CoP) were recorded throughout the experiment. The EEG was recorded with 32 active Ag–AgCl electrodes (EasyCap GmbH, Herrsching, Germany) distributed across the scalp according to the international 10–20 system [26], using a wireless amplifier (Siesta, Compumedics Neuroscan, Australia) and the Profusion EEG software (Compumedics Neuroscan, Australia). The sampling rate was 512 Hz. Before each walking block, EEG was recorded for 2 min during quiet stance. To reduce potential artifacts in the EEG, the participants were instructed to limit their head movements and, to avoid talking, and excessive blinking.

The GRF and CoP for each foot were separately recorded with two force plates embedded in the treadmill (M-Gait, Motekforce Link, Netherlands). These data were recorded with D-Flow 3.26.0 (Motekforce Link, Netherlands) with a variable sampling frequency (later resampled at 300 Hz). A digital trigger was simultaneously recorded by both systems (Profusion and D-Flow) for synchronization.

### Data analysis

All analyses were performed using MATLAB version 2014b (The MathWorks Inc., USA) with the addition of EEGLAB 14.1.2b (Swartz Center for Computational Neuroscience, USA) for EEG analyses.

#### Gait cycle segmentation

The gait events for heel strike and toe off were extracted from the GRF via threshold detection. The GRF data were filtered with a zero-lag low-pass 4th order Butterworth filter (10 Hz) and compared against a force threshold set to 30 N. Heel strike events were detected when the GRF exceeded the force threshold. Similarly, toe off events were detected when the GRF dropped below the force threshold. The gait events were aligned with the EEG using the digital trigger for synchronization.

#### Gait parameters

To assess any modifications of the gait pattern, the following gait parameters were computed: gait cycle duration, stance phase duration, step width, maximal GRF, and the GRF trace over time. The *gait cycle duration* was defined as the time difference between consecutive right heel strikes. The *stance phase duration* was defined as the percentage of the gait cycle spent between heel strike and toe off from the same foot. The *step width* was defined as the mediolateral distance of the filtered CoP (zero-lag, band-pass 4th order Butterworth filter, 0.5–15 Hz) between both feet during the double support phase of the gait cycle. The *maximal GRF* was defined through each gait cycle and for each foot, and it was normalized by the participant’s bodyweight (in Newton). The GRF trace was segmented according to the gait cycles of the right foot and time-normalized for gait cycle duration and the following fixed gait events: heel strike right (0%), toe off left (12%), heel strike left (50%), toe off right (62%) and heel strike right (100%). For group-level analyses, the mean of each parameter (gait cycle duration, stance phase duration, step width, maximal GRF, and the GRF trace over time) was computed (per participant) over all gait cycles within each walking condition.

#### EEG analysis

A schematic overview of the EEG processing steps can be found in Additional file 1. This approach is in line with previous studies on cortical dynamics during whole-body movement [15, 20, 27]. During acquisition, the EEG was filtered with a notch filter (50 Hz) to remove line noise. After acquisition, the EEG was filtered with a zero-phase high-pass FIR filter (1 Hz) and further processed with the CleanLine EEGLAB plugin [28, 29] to reduce line noise harmonics (100 and 150 Hz). The EEG was visually inspected for artifacts and noisy channels. Only one channel was removed in two of the participants.

The EEG was re-referenced to the common average and processed with the artifact subspace reconstruction (ASR) EEGLAB plugin that was used [30] to automatically remove non-stationary large-amplitude artifacts from the data. During a calibration stage, the ASR method determines a noise-free subspace from the continuous data, via principal component analysis (PCA). Then, a sliding-window PCA is computed over the data and compared against the noise-free subspace. If the variance of any principal component is above a certain threshold, the principal component is labeled as artifact and removed from the data. To ensure proper calibration of the ASR, the quiet stance EEG data were appended to the experimental data recorded during the walking blocks. The ASR user interface was configured to remove channels if the correlation with surrounding channels was less than 0.5, to reconstruct artifacts lying beyond ten standard deviations from the calibration data, and to remove a 500 ms time window from all channels if more than 25% of the channels contained artifacts at that moment in time.

After preprocessing, the EEG data were segmented into epochs ranging from − 0.4 to 2.2 s surrounding the right heel strike (i.e., the side of the dummy prosthesis). Epochs which did not contain a standard sequence of gait events (heel strike right, toe off left, heel strike left, toe off right, and heel strike right) were removed. Epochs with flat lines were visually identified and removed from the individual EEG datasets. The average number of remaining epochs (gait cycles) for the walking without dummy prosthesis, first, and last time walking with dummy prosthesis were (mean ± SD) 187 ± 13, 126 ± 26, and 146 ± 24, respectively.

#### Source separation

The segmented EEG data were separated into components from independent brain sources using Infomax independent component analyses (ICA) [31–34]. Then, the variance of individual epochs was computed for each independent component (IC) and normalized using the z-score per component across all epochs. Epochs with a normalized variance exceeding three standard deviations were marked as artifacts and removed from the data (resulting in 181 ± 14, 122 ± 26, and 141 ± 25 epochs remaining for walking without, first, and last time walking with dummy prosthesis). Afterwards the ICA was recomputed to ensure components were based on artifact-reduced EEG data. The resulting ICs were associated with an equivalent current dipole using a standardized three-shell boundary element head model (Montreal Neurological Institute (MNI)) and standard electrode positions (EEGLAB plugin DIPFIT; [35]). ICs were identified as possible brain sources according to their anatomical location (inside the head volume) and when residual variance of their equivalent current dipole was < 15% (mean number of ICs per participant: 3 ± 1.7, range 1–8).

The selected components were clustered across participants using the k-means clustering algorithm (k = 3) based on the following features: 3D anatomical location of their equivalent current dipoles, their mean power spectral density (PSD) (frequency band 3–48 Hz), their associated scalp projection, and their mean spectrogram across trials. These features were reduced to 10 principal components before clustering. Equivalent current dipoles (ECDs) which were located more than three times the standard deviation of distances within a cluster from any cluster centroid were considered outliers and were removed. Clusters with ECDs of at least half of the participants (n ≥ 6) were kept for statistical analysis. When clusters contained multiple ECDs of one participant, a single ECD with the shortest distance to the cluster centroid was retained for analysis. The Yale BioImage Suite [36] was used to determine the location of the cluster centroid and its corresponding Brodmann area.

#### Event-related spectral perturbation time–frequency maps

Event-related spectral perturbation (ERSP) time–frequency maps were used to compute modulations of intrinsic cortical rhythms [31, 37]. From each epoch (i.e., one gait cycle), single-trial spectrograms were computed and time-warped to normalize the duration of the gait cycle across all walking conditions.^1^ The gait cycle and the gait events onset (i.e., heel strike right, toe off left, heel strike left, toe off right, and heel strike right) were normalized, using linear interpolation, to the median gait cycle duration and event onsets across all participants, all conditions, and all steps.

Average log-transformed spectrograms showing relative power changes were computed per individual IC and walking condition as the average difference between each (log-transformed) single-trial spectrogram and the average (log-transformed) spectrogram from the entire epoch (baseline). For visualization purposes, condition-specific baselines (i.e., the log-transformed power spectrum) were obtained from each walking condition over the complete gait cycle duration. Average time–frequency maps for a given IC cluster were computed by averaging across the maps corresponding to the ICs that were members of the cluster, separately for each walking condition.

### Group-level statistical analyses

#### Gait parameters

Statistical analyses for the gait cycle duration, stance phase duration, step width, and maximal GRF were done with IBM SPSS Statistics 25 (IBM B.V., the Netherlands). The normal distribution of each parameter was first checked with the Shapiro–Wilk test. A repeated measures one-way ANOVA with post-hoc tests was conducted if the distribution was normal, otherwise a Friedman test was conducted with Wilcoxon signed rank tests for post-hoc testing. All post-hoc tests were Bonferroni corrected. The significance level for all tests was set at α = 0.05.

For the statistical analyzes of the GRF trace, MATLAB was used. A repeated measures one-way ANOVA was conducted of the normalized and time-warped GRF within the gait cycle. The significance level was set at α = 0.05 and it was corrected for false discovery rate [38] due to the multiple tests over the individual time points. Post-hoc comparisons were conducted with paired two-tailed t-tests and corrected in the same way.

#### Event-related spectral perturbation

MATLAB was used for comparison of the ERSP maps between conditions. The ERSP maps for the three walking conditions were computed with a common baseline (log-transformed spectrogram of walking without the dummy prosthesis). The significance of the modulations of cortical rhythms was determined with non-parametric permutation statistics [39, 40]. First, a one-way ANOVA of the ERSP maps with three levels (i.e., the walking conditions) was computed, and the resulting F-statistic per time point was stored. Then, a surrogate random distribution was created through random permutations of the condition labels (n = 200), followed by calculation of the surrogate F-statistic. The significance of the original F-statistic was determined by comparing against the surrogate distribution (critical alpha α = 0.05). Post-hoc tests (paired two-tailed t-tests) were conducted in a similar way. The significance level was corrected for false discovery rate [38].

## Results

### Gait parameters

**Fig. 2:**
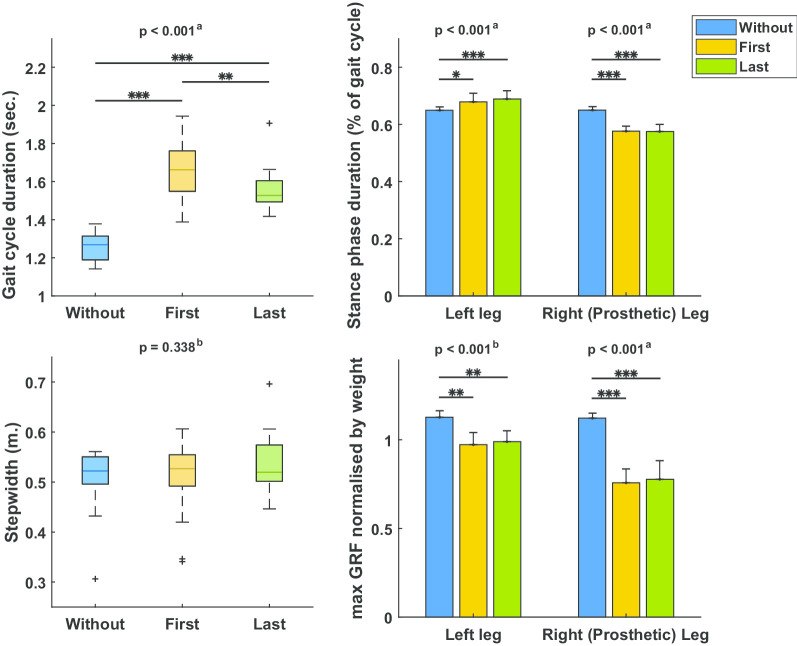
Gait parameters from walking without, first time and last time walking with a dummy prosthesis. ^a^One-way repeated measures ANOVA, ^b^Friedman’s ANOVA, *p < 0.05, **p < 0.01, ***p < 0.001

Figure 2 shows the mean and standard deviation of the gait cycle parameters. Gait cycle duration (F(2,22) = 112.3, p < 0.001), stance phase duration of the right (prosthetic) (F(2,22) = 98.4, p < 0.001) and left leg (F(2,22) = 16.0, p < 0.001), and the maximal GRF of the right (prosthetic) (F(2,22) = 135.5, p < 0.001) and left leg (χ^2^(2) = 18.7, p < 0.001) all differed between blocks. Only step width did not differ between blocks (χ^2^(2) = 2.2, p = 0.338).

Post-hoc testing was done between the three different condition. During the first time walking, in comparison to walking without the dummy prosthesis, the gait cycle duration was significantly longer, the stance phase duration was significantly shorter for the right (prosthetic) leg, and significantly longer for the left leg, and maximal GRF was significantly lower for both legs. Similar effects were found during the last time walking in comparison to walking without dummy prosthesis. No significant difference was found between the first and last time walking with the dummy prosthesis in the stance phase duration, and in the maximal GRF for both the right (prosthetic) and left leg. Differently, gait cycle duration was significantly shorter in the last time walking in comparison to the first time walking with dummy prosthesis.

**Fig. 3:**
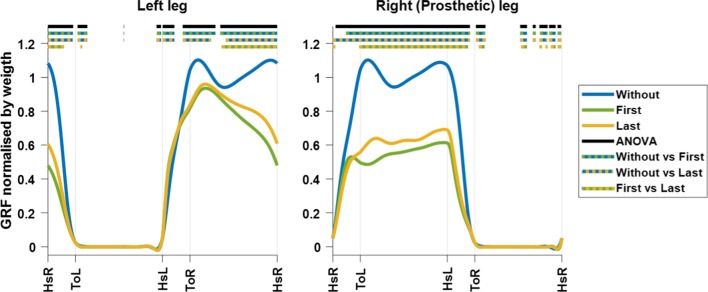
The GRF trace over the gait cycle. The GRF trace is shown for the left and right leg during walking without, first time and last time walking with a dummy prosthesis. The lines below show at which time point there is a significant difference (p < 0.05). The black line shows the significant difference between all the conditions (ANOVA). The dotted lines represent the significant difference between the two conditions, which colours are represented

Figure 3 shows the GRF trace over the gait cycle. Significant differences in the GRF trace between all blocks occurred during the stance phase of either leg. For the right (prosthetic) leg, the GRF during the stance phase was significantly lower in first and last time walking with the dummy prosthesis in comparison to walking without. During the last time walking with dummy prosthesis, the GRF was significantly higher during mid stance in comparison to the first time walking with the dummy prosthesis. For the left leg, it can be seen that the GRF peak during early stance is significantly lower during first and last time walking with the dummy prosthesis in comparison to walking without. The GRF peak late stance was significantly lower during the first time walking compared to walking without dummy prosthesis, and significantly higher during the last compared to the first time with dummy prosthesis.

### Clusters of independent components

**Fig. 4:**
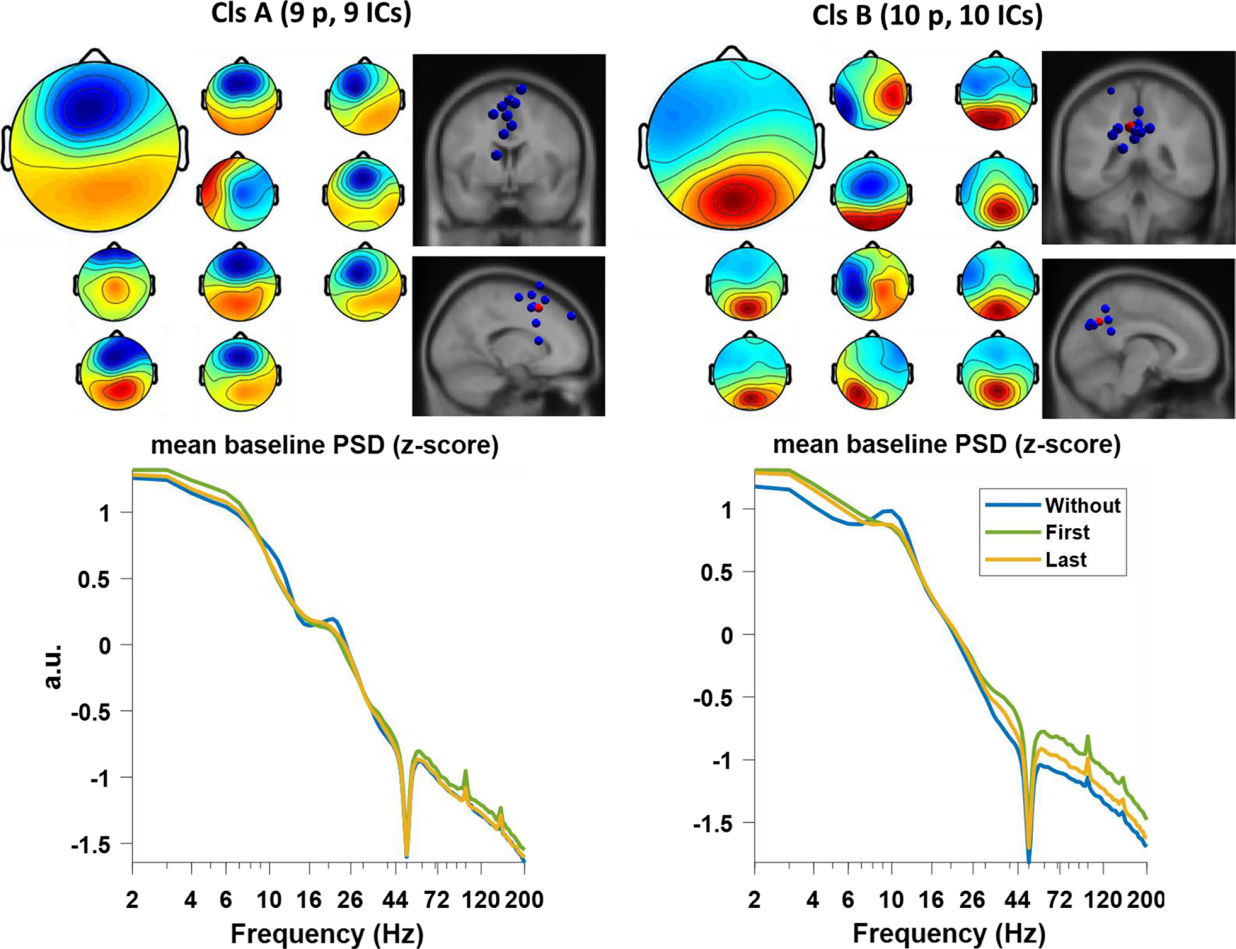
Scalp projections, dipole locations and mean PSD of the two clusters. The mean PSD is of the baseline for each condition. According to the location of the cluster centroid, Cluster A is specified as fronto-central and cluster B is specified as the parietal. P stands for the number of participants in the cluster and IC for the number of independent components. The scalp projection of the cluster (larger image) and of each individual component (smaller images) are presented. The blue dipoles represent the location of individual independent components and the red dipoles represent the cluster centroid

Two clusters with independent components from more than half of the participants were found. Figure 4 shows the location of the corresponding equivalent current dipoles. The MNI coordinates of the cluster centroids were Cluster A {x = − 2, y = 17, z = 45} (Brodmann area 8, fronto-central cluster) and Cluster B {x = − 8, y = –58, z = 37} (Brodmann area 31, parietal cluster) with ICs from 9/12 and 10/12 participants, respectively. These coordinates provide an approximation to the localization of the actual cortical sources, limited by the spatial resolution of the source localization methods (standard electrode positions and standard head model).

### Modulations of cortical rhythms within the gait cycle

**Fig. 5:**
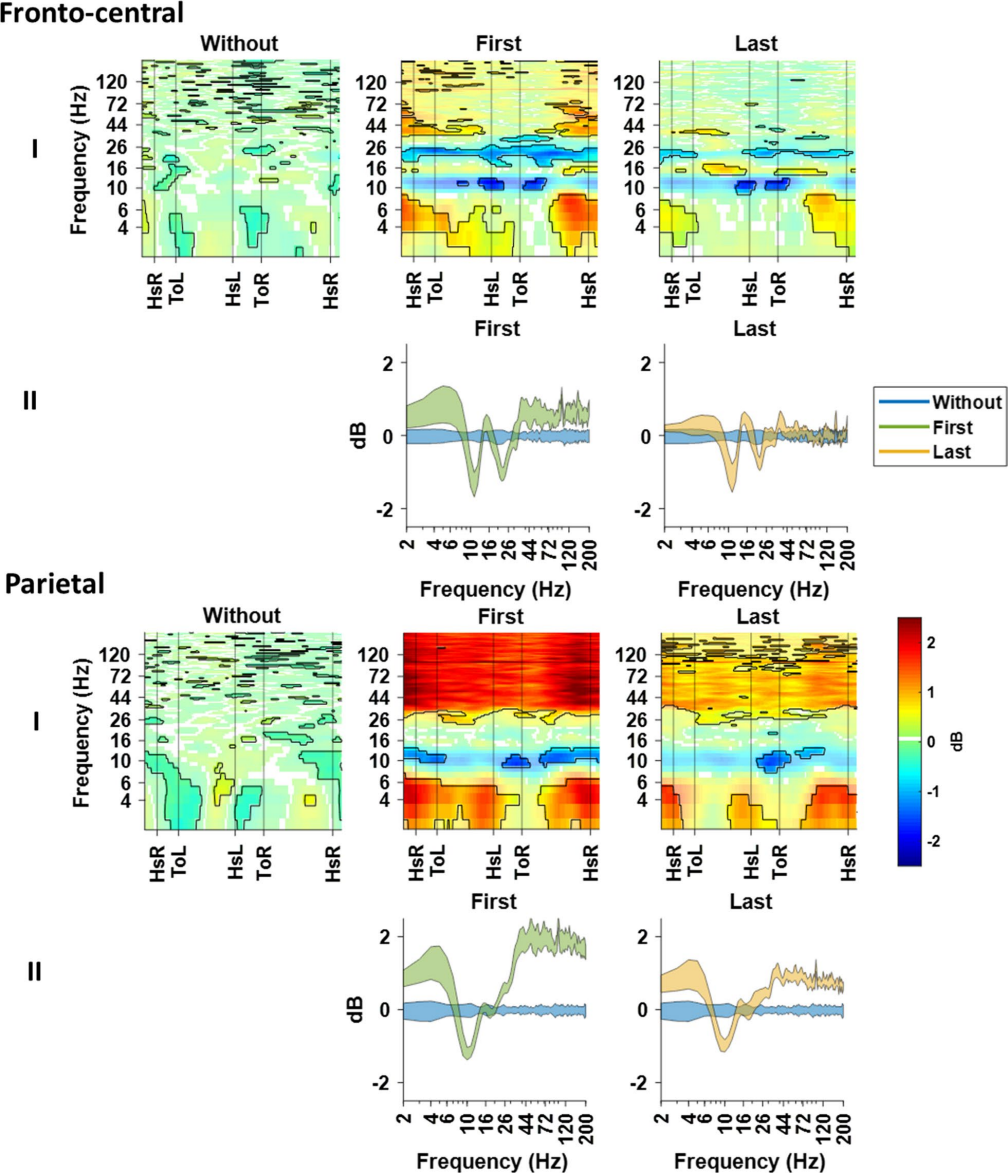
Event-related spectral perturbations time–frequency maps and mean ERSP across the gait cycle. The ERSP(I) and mean ERSP(II) are displayed for the fronto-central and parietal clusters. I: Time–frequency maps show the decrease (blue) and increase (red) in mean power for each condition, relative to the baseline of walking without dummy prosthesis. The non-significant differences from this baseline are partially masked with a white overlay. II: The mean ERSP across the gait cycle shows the power increase (positive) or decrease (negative) in the mean power and standard deviation over frequencies. The blue shaded region represents the power of walking without dummy prosthesis (baseline), green represents first time and yellow represents last time walking with dummy prosthesis

Figure 5 shows the mean time–frequency maps with a common baseline of walking without dummy prosthesis. For the fronto-central cluster, the mu (9–13 Hz) and beta (20–26 Hz) band show power decrease during the first and last time walking with the dummy prosthesis in comparison to walking without (Fig. 5, fronto-central). In addition, power increase can be seen in theta (3–7 Hz) and gamma (34–44 Hz) bands, which appears to be most pronounced during the first time walking in comparison to walking without dummy prosthesis (Fig. 5, fronto-central). For the parietal cluster, power increases are found in the theta (3–7 Hz) and gamma (35–120 Hz) band in the first and last time walking with dummy prosthesis (Fig. 5, parietal), whereas the power increase of gamma seems to be smaller in the last time in comparison to the first time walking with the dummy prosthesis (Fig. 5, parietal II). Further, in the mu (9–13 Hz) band power modulations are decreased during first and last time walking with the dummy prosthesis.

Further analyses were done to determine the modulations of cortical rhythms within the gait cycle. Based on the results displayed in Fig. 5, the power modulations within the gait cycle of the fronto-central and parietal clusters were analysed for the theta (3–7 Hz), mu (9–13 Hz), and gamma bands (34–44 Hz, 35–120 Hz, respectively). For the fronto-central cluster, the power modulations within the gait cycle of the beta band (20–26 Hz) were additionally analysed.

**Fig. 6:**
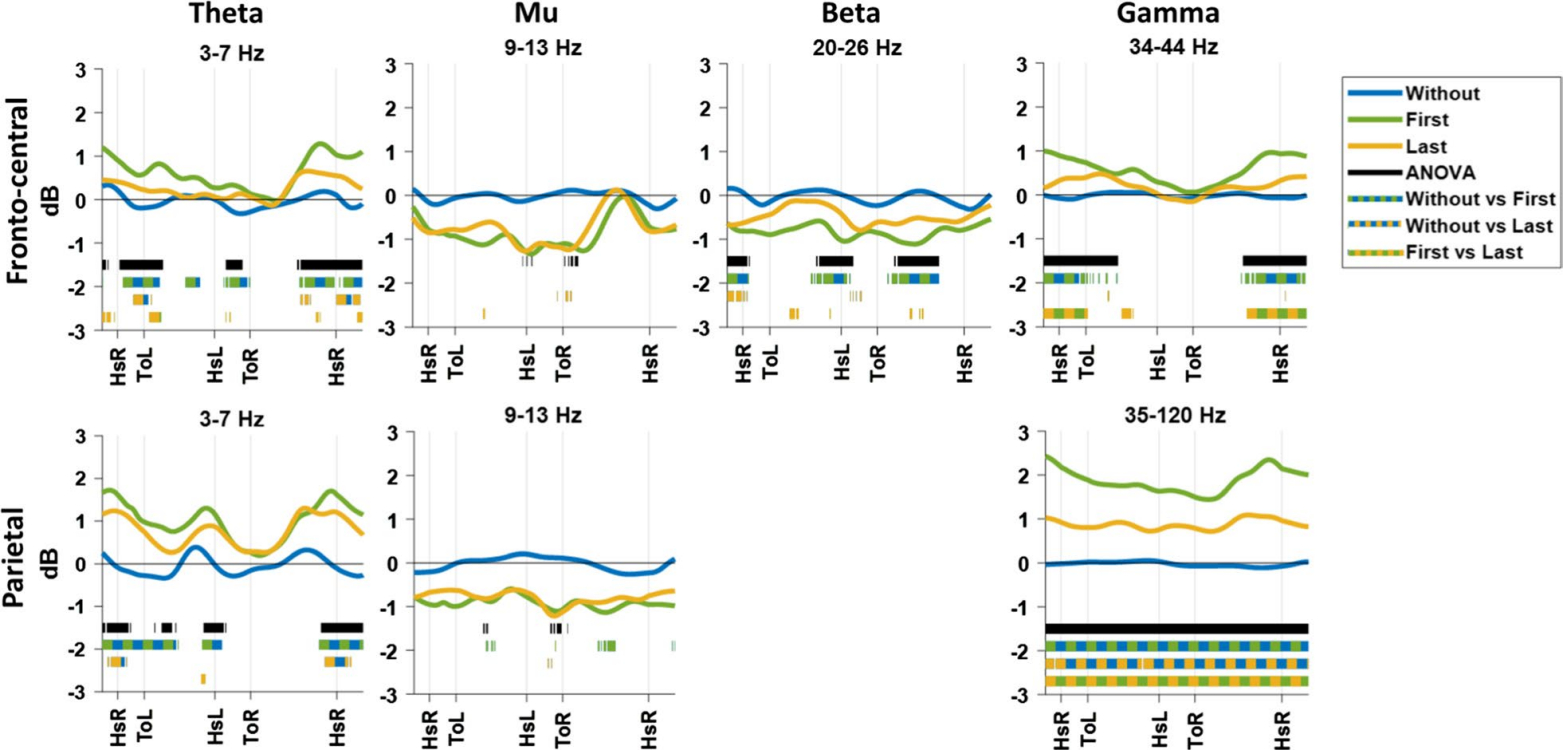
The mean power modulation over time across different frequency bands. Blue line represents walking without dummy prosthesis, green represents first time and yellow represents last time walking with dummy prosthesis. The black line indicates at which time point there is a significant difference between all the conditions (ANOVA). The dotted lines represent the significant difference between the two conditions, of which the colours are represented

Figure 6 shows power modulations within the gait cycle, for the frequency bands specified in the previous section. For the fronto-central and parietal cluster, the theta band shows increased power around heel strike and weight acceptance on the right (prosthetic) leg. Similarly, theta band modulation in the parietal cluster appears around heel strike of the left leg. In the mu band of the fronto-central and parietal cluster, a brief time period around toe off of the right (prosthetic) leg shows a significant decrease in power for the last time walking with in comparison to walking without dummy prosthesis. The beta band in the fronto-central cluster shows significant differences during the swing phase of the right (prosthetic) leg and around heel strike of both legs, where the power of the first time walking with is significantly decreased compared to walking without dummy prosthesis. The gamma band power from the fronto-central cluster shows a significant increase around heel strike of the right (prosthetic) leg in the first time walking compared to walking without. This increased power shows a significant decrease in the last time compared to the first time walking with the dummy prosthesis. A similar pattern appears for the power of the gamma band from the parietal cluster, but no significant gait-cycle dependent modulations could be identified due to the significantly increased offset for first and last time walking with the dummy prosthesis in comparison to walking without. This offset in power of the gamma band during the last time walking with dummy prosthesis is significantly decreased compared to first time walking with dummy prosthesis.

## Discussion

In this study modulations of cortical rhythms that accompany short-term gait modifications were identified during first-time use of a dummy mechanical knee prosthesis. Able-bodied individuals walked with a dummy prosthesis for their first time, in a short experimental session. All gait parameters (stance phase duration, step width, maximal GRF, and the GRF trace over time) revealed immediate modifications of the gait pattern when participants were introduced to the dummy prosthesis. Except for the gait cycle duration, these modifications remained throughout the duration the experimental session. Interestingly, power modulations of the theta, mu, beta, and gamma rhythms, source-localized from scalp-level recordings to the fronto-central and the parietal cortical regions, accompanied the modifications of the gait pattern throughout the experimental session. These power modulations of the theta, beta, and gamma rhythms within the gait cycle differed between walking conditions.

Gait modifications occurred from the first experimental block walking with the dummy prosthesis. In comparison with walking without the dummy prosthesis, the duration of the gait cycle increased in the first experimental block and was slightly reduced in the last experimental block (after roughly 12 min of practice). Additionally, the participants modified their gait pattern to spend less time supporting themselves with the dummy prosthetic leg and decreased their body weight support while standing on either leg. The changes in stance phase duration and body weight support suggest reduced confidence in the support of the dummy prosthetic leg and increased use of the handrails to support the body weight during the gait cycle. The use of the handrails for support while standing on the dummy prosthesis is clearly seen in the trace of the GRF (Fig. 3). Moreover, the GRF trace indicates consistent use of the handrails for weight support with either leg, showing a reduction of the amount of support through the experimental session. These results indicate short-term gait modification related to the use of the dummy prosthesis, that are sustained through an initial experimental session.

Together with the gait modifications, power modulations of cortical theta, mu, beta, and gamma rhythms were found, presumably originating from the fronto-central (centroid of cluster A) and parietal cortical regions (centroid of cluster B), according to the source localization analysis. In previous studies, the involvement of fronto-central and parietal cortices during gait adaptation has been related to top-down control (e.g., motor planning and inhibitory control; fronto-central), and integration of sensorimotor feedback (parietal) [13, 15]. Consistent with these previous reports, a power decrease of the mu rhythm from the fronto-central and parietal cluster during walking with the prosthesis was found compared to walking without dummy prosthesis. The decrease in power of the mu rhythm occurs around toe off of the prosthetic leg. During this phase of the gait cycle, it is estimated how much clearance should be provided between the prosthetic foot and the ground, and how much active swing force should be provided to the prosthetic leg, which together determine the ability to successfully complete the swing phase of the prosthetic leg. Therefore, the power decrease of the mu rhythm may indicate increased motor planning and sensorimotor integration during the pre-swing with the prosthetic leg.

The results also showed a power decrease of the beta rhythm from the fronto-central cluster during the swing phase of the prosthetic leg and heel strike of both legs. A power decrease of the beta rhythm from the fronto-central cluster during gait adaptation has previously been related to increased cognitive control [15, 41]. During the swing phase of the prosthetic leg, enough clearance should be provided between the prosthetic foot and the ground to avoid a stumble or trip. Furthermore, a correct placement of the prosthesis during heel strike ensures the prosthetic knee to lock so it can be safely loaded during stance phase. As these events in the gait cycle are essential to avoid a stumble or trip, the power decrease of beta rhythm from the fronto-central cluster probably indicates additional cognitive load for top-down control of the prosthesis during swing phase and the placement of the prosthesis during heel strike. Importantly, the use of handrails to support the body weight must be taken into account. During right heel strike, the body weight is transferred to the prosthetic leg, but also to the arms, as can be seen in the GRF trace. This shift in body weight could also require increased top-down control and therefore contribute to the power decrease of the mu (fronto-central and parietal) and beta (fronto-central) rhythms.

In addition to these results, a power increase was found in theta rhythms in the fronto-central and parietal clusters around the heel strike of both limbs. In previous studies, a transient power increase of the theta rhythms from a fronto-central cortical region has been related to the control of balance and posture [42] and the monitoring of postural stability during quiet stance [43–45] and walking [46]. Additionally, power increase of the theta rhythm from posterior-parietal cortical regions has been related to error detection during movement and the mismatch between intended action and sensory feedback [20, 47]. During walking with a dummy prosthesis, the heel strikes with the dummy prosthesis might feel unusual and unstable, as the prosthesis is an external walking aid attached to the body. The use of the prosthesis creates new sensory input during walking, in addition to an altered perception of postural stability that comes with this modification. The power increase of the theta rhythm occurring around heel strike might therefore indicate an increase in sensorimotor processing and the assessment of postural stability during stepping.

During the whole gait cycle, the power of the gamma rhythm from the parietal cluster is increased, especially around heel strike of the prosthetic leg. Power modulations of the gamma rhythm have been related to goal directed behavior in visual search tasks [48] and to initial visual motor learning during gait adaptation [49]. Here, the visual feedback from the placement of the prosthetic foot might be used to anticipate the prosthesis behavior and modify the gait pattern when needed, which could cause the power increase of the gamma band in the parietal cluster. In addition to the power increase of the theta rhythm, the results reveal a power increase of gamma rhythms from the parietal cluster during first walking with the prosthesis, which both (theta and gamma) subsequently diminish over the period of the experimental session. Previous research reported that power increase of the gamma rhythm could be related to the power increase of the theta rhythm via cross-frequency coupling [50]. The theta-gamma cross-frequency coupling has been associated with short-term memory processing [50–52], which may indicate a learning process of new motor memories [49, 53]. Hence, the current findings may indicate that new motor memories are being formed during walking with the prosthesis and that during the experimental session progress might have been made in forming these memories. Further analysis that specifically target cross-frequency coupling are needed to corroborate this observation.

When interpreting these results, several limitations need to be considered. First, the spatial resolution of our analysis is limited by the use of multichannel (32 channels) EEG, standard electrode locations and standard head models. However, although the spatial resolution of the source localisation for each component is limited, the group-level analysis (represented by the cluster centroid) may provide a more accurate estimation of the cortical source location. For this reason, our interpretations are restricted to the likely cortical regions where the cluster centroids are located. Previous studies on cortical contributions to gait control often reported modulations of rhythmic activity from fronto-central and parietal cortical areas; in particular from supplementary motor are (Brodmann area 6) and the posterior parietal cortex (Brodmann area 7). Importantly, these cortical locations are adjacent to the estimated locations reported in this study and their functional mapping does overlap [54, 55]. Therefore, the interpretation given here is consistent with our current understanding of the cortical gait control. Second, despite the measures taken during data acquisition and the careful artefact-correction and -rejection, some artefacts may still be present in the EEG data. Thus, caution must be exercised when interpreting broad modulations of the gamma rhythm in parietal and occipital regions, as complete removal of spurious EMG activity cannot be completely achieved. Notably, the PSD of the fronto-central and parietal clusters (see Fig. 4) do not suggest a strong impact of EMG, as the PSDs follow a typical 1/f power decay characteristic for oscillatory cortical activity. Third, our pilot study is limited by its sample size (n = 12), and therefore generalizing the results cannot be granted. Yet, taking into consideration the sample size of previous EEG studies (ranging from 4 to 37 participants) [10, 13, 15, 17, 19, 20, 27, 30, 41–44, 46, 48, 49, 52, 56], the sample size of the current pilot study is acceptable.

Importantly, we present a first step towards identifying the cortical modulations of short-term gait modifications during walking with a prosthesis; yet, to further investigate the neural mechanisms of walking with a prosthesis, the neural pathways involved in these modifications must be identified. This could be done by defining the interactions between the central and peripheral nervous system using for instance (effective/directed) corticomuscular coherence [19, 56]. Future studies should strive to acquire high-density (100+ channels) EEG and person specific electrode locations to improve the source localisation. Furthermore, a close inspection of data quality (against severe movement artefacts) and integrity (e.g., against sample loss and flat lines) needs to be conducted during data acquisition.

## Conclusion

Immediate gait modifications to the use of a prosthesis are accompanied by modulations of the mu (fronto-central and parietal cortical regions) and beta (fronto-central) rhythms, as well as theta and gamma rhythms (fronto-central and parietal). The modulations of cortical activity could be related to whole-body coordination, including the swing initiation and the placement of the prosthesis, as well as the bodyweight transfer between legs and arms. During a short experimental session limited gait adaptation could take place, as indicated by multiple gait parameters. The observed power modulations of the gamma rhythm may indicate that an initial motor memory of the new gait pattern is formed within the duration of a short (12 min) experimental session. To our knowledge, this is the first study to show the cortical mechanisms of short-term adaptation of able-bodied individuals to walking with a dummy prosthesis. Future efforts will focus on determining the effects of long-term adaptation on cortical modulations and its neural correlates.

## Supplementary information

**Additional file 1.** Schematic overview of the EEG data processing.

## Data Availability

The datasets used and/or analyzed during the current study are available from the corresponding author on reasonable request.

## Abbreviations

ASR: Artifact subspace reconstruction
CoP: Center of pressure
ECD: Equivalent current dipoles
EEG: Multi-channel electroencephalogram
ERSP: Event-related spectral perturbation
GRF: Ground reaction force
IC: Independent component
ICA: Independent component analyses
MNI: Montreal Neurological Institute
PCA: Principal component analysis
PSD: Power spectral density

## Footnotes

1 Via the optional parameter 'timewarpms' in the newtimef.m function from EEGLAB.
