# Supplementary figures and images for: Rhythmic neural activity is comodulated with short-term gait modifications during first-time use of a dummy prosthesis: a pilot study
Source: J Neuroeng Rehabil. 2020 Oct 8;17:134. doi: 10.1186/s12984-020-00761-8 (PMC7542708; doi:10.1186/s12984-020-00761-8)

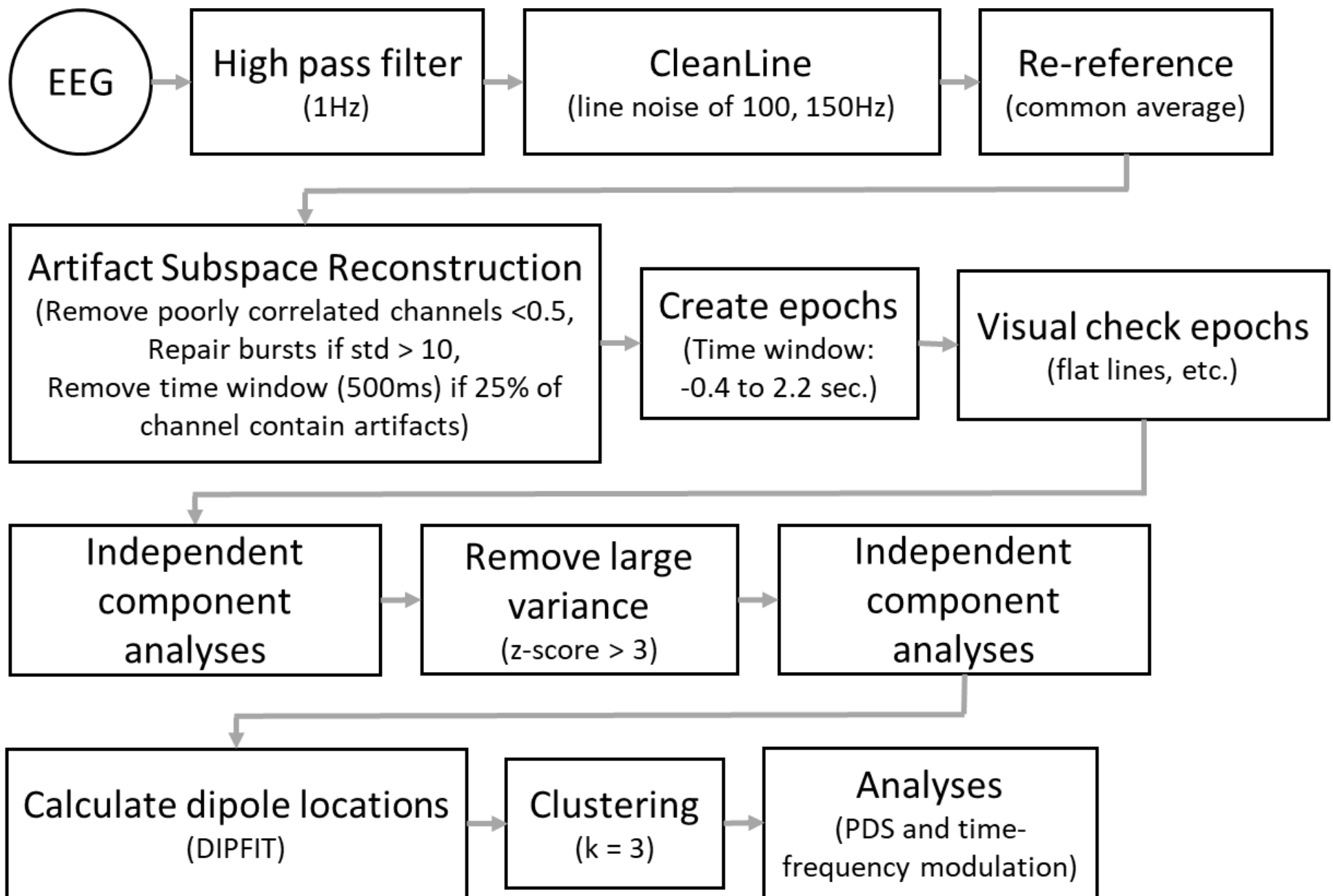

Supplement: Supplementary file 1 — Additional file 1. Schematic overview of the EEG data processing. [file 12984_2020_761_MOESM1_ESM.pdf]
